# DiLFM: an artifact-suppressed and noise-robust light-field microscopy through dictionary learning

**DOI:** 10.1038/s41377-021-00587-6

**Published:** 2021-07-27

**Authors:** Yuanlong Zhang, Bo Xiong, Yi Zhang, Zhi Lu, Jiamin Wu, Qionghai Dai

**Affiliations:** 1 Department of Automation, Tsinghua University, Beijing, 100084 China; 2 Institute for Brain and Cognitive Sciences, Tsinghua University, Beijing, 100084 China; 3 Beijing National Research Center for Information Science and Technology, Tsinghua University, Beijing, 100084 China

**Keywords:** Microscopy, Biophotonics

## Abstract

Light field microscopy (LFM) has been widely used for recording 3D biological dynamics at camera frame rate. However, LFM suffers from artifact contaminations due to the illness of the reconstruction problem via naïve Richardson–Lucy (RL) deconvolution. Moreover, the performance of LFM significantly dropped in low-light conditions due to the absence of sample priors. In this paper, we thoroughly analyze different kinds of artifacts and present a new LFM technique termed dictionary LFM (DiLFM) that substantially suppresses various kinds of reconstruction artifacts and improves the noise robustness with an over-complete dictionary. We demonstrate artifact-suppressed reconstructions in scattering samples such as *Drosophila* embryos and brains. Furthermore, we show our DiLFM can achieve robust blood cell counting in noisy conditions by imaging blood cell dynamic at 100 Hz and unveil more neurons in whole-brain calcium recording of zebrafish with low illumination power in vivo.

## Abstract (web summary)

Dictionary light-field microscopy (DiLFM) substantially eliminates various kinds of reconstruction artifacts and improves the noise robustness. Using DiLFM yields accurate observation of zebrafish blood flow at 100 Hz in low-light conditions.

## Graphical abstract

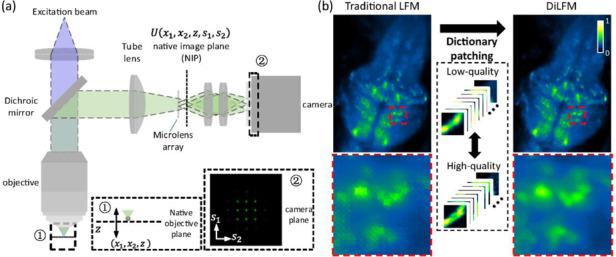

## Introduction

Cellular motions and activities in vivo are usually in millisecond time-scale and in 3D space, including voltage and calcium transients of neurons^1,2^, blood cell flows in beating hearts^3^, and membrane dynamics in embryo cells^4^. Observing and understanding these fantastic phenomena requires abilities to record cellular structures with a high spatiotemporal resolution in 3D. Many techniques are developed to meet this requirement, including confocal^5,6^ and multiphoton scanning microscope^7^, selective plane^8^, and structured illumination microscopy^9^. Although these techniques can access the 3D structures in combination with depth scanning, the temporal resolution is limited by the inertia of the scanning devices or the single-plane recording rate. Therefore, a number of advanced techniques with multiplexing techniques and optimized sampling strategies have been introduced, such as multiplane or multifocal imaging^10^, scanning temporal focusing microscopy^11^, and random access microscopy^12^. However, heat tolerance of living animals or organs and sample density still prevent those methods to achieve high throughput with low light doses.

Light field microscopy (LFM) emerges as a popular tool in incoherent imaging of volumetric biological samples within a single shot^13–19^. This is achieved by capturing a 4D light field on a single 2D array detector through specific optical components such as the microlens array (MLA). The 3D information of biological samples is extracted from 4D light field measurements through multiple Richardson-Lucy (RL) reconstruction iterations^20,21^. The lack of a scanning device makes LFM a high-speed volumetric imaging tool for biological systems, with various applications in live-cell imaging^18^, volumetric imaging of beating hearts and blood flow^22^, and neural recording^23,24^, to name a few. Although LFM has achieved great success, current LFM implementations suffer several disadvantages: (1) inherent trade-offs between improving reconstruction contrast and reducing ringing effects at edges; (2) severe block-wise artifacts near the native image plane (NIP)^13^; (3) contaminations to 3D-resolved structures from depth crosstalk; and (4) quick performance degradation under low single-to-noise ratio (SNR) situations. The reasons for these drawbacks are due to low spatial sampling and illness of restoring 3D information from 2D sensor images.

Many methods are proposed to mitigate parts of these drawbacks. To avoid NIP artifact, it is straightforward to carefully set the imaging volume on one side of the NIP^23^, which reduces the imaging depth range a lot. Shifting the MLA to avoid NIP will sacrifice the depth-of-field^18^. Methods that reshape PSF^25^ or add additional views^22,26^ can help ease edge ringing and improve the contrast, but either require customized optical components or complicate the system by adding scanning devices or more objectives. The additional complexity of adding more hardware also hampers the usage of LFM in freely behaving animals for volumetric functional imaging due to space and weight limitation^27^. On the other hand, modifying the reconstruction algorithm to mitigate the LFM artifacts is more convenient and flexible since adjusting the hardware is avoided. Introducing a strong blur in the reconstructed volume can reduce the NIP block-wise artifacts and depth crosstalk, but the imaging resolution will be much worse^28^. The phase space reconstruction approach by Zhi et al. achieves faster convergence and reduces NIP block-wise artifacts through serially reconstructing different angular views^29^ but cannot solve the contrast and ringing dilemma. All these implements suffer from noise-induced artifacts when imaging phototoxicity-sensitive samples like mitochondria and zebrafish embryos.

**Fig. 1:**
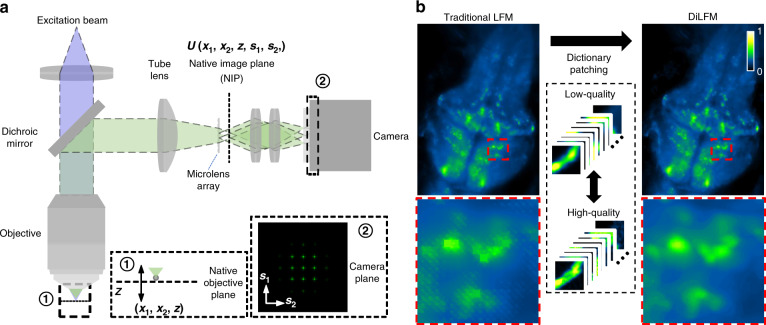
Principle of DiLFM. **a** Light field microscopy (LFM) imaging scheme. 3D distributed samples will be collected by standard microscopy (i.e., an objective with a tube lens), then coded by a microlens array (MLA) and captured by a camera. Zoom-in panel ① shows the relationship of a sample space point with the pattern in the native objective plane (NIP). Zoom-in panel ② shows an exemplary LFM PSF captured by the camera. **b** Principle of DiLFM. After ringing-reduced RL reconstruction, DiLFM replaces low-quality image elements with high-quality ones to suppress reconstruction artifacts

Here, we propose a new LFM method based on dictionary patching, termed DiLFM, to enable fast, robust, and artifact-suppressed volumetric imaging under different noisy conditions without hardware modifications (Fig. 1). Our approach is motivated by recent results in sparse signal representation, suggesting that artifact-free signals can be well represented using a linear combination of few elements from a redundant dictionary even under heavily noisy conditions^30^. The systematic artifacts due to the low sampling rate in LFM can be compensated by dictionary priors learned from general biological samples. Our DiLFM reconstruction is a combination of a few RL iterations to provide basic but ringing-reduced 3D volumes with a dictionary patching process to fix the reconstruction artifacts and improve the resolution and contrast (Fig. S1). We train a pair of low- and high-fidelity dictionaries under LFM forward model such that only matched low-fidelity snippets from RL reconstructions will be updated by high-fidelity elements. With the robustness of both few-run RL and dictionary patching in low-SNR conditions, our DiLFM provides superior performances over other methods under noise contaminations. We demonstrate the contrast improvement and artifacts reduction by DiLFM via multiple simulations and experiments, including the *Drosophila* embryo and brain. We show the robustness of DiLFM in observing zebrafish blood flow at 100 Hz in low-light conditions. We further demonstrate that our DiLFM enables finding two times more neurons in zebrafish brain in vivo with low-power illumination.

## Results

### DiLFM substantially suppresses artifacts in LFM

Due to the illness of LFM reconstruction, traditional LFM usually generates several kinds of artifacts with RL deconvolutions (Methods and Fig. S1). First, we find traditional LFM cannot achieve high contrast and ringing-suppressed reconstructions at the same time. We numerically simulate a USAF-1951 resolution target at *z* = −50 µm and find the core of the square becomes dimmer and dimmer as more RL iterations are involved (Fig. S2a, b). The intensity cross-section of an originally sharp edge clearly shows a ringing feature, while the peak-to-valley ratio increasing with the iteration number suggests improved contrast (Fig. S2c–e). We argue that such contrast increase is at the cost of image structure distortions, which should be alleviated for quantitative analysis. Another well-known artifact of LFM is the block-wise features in NIP due to the insufficient spatial sampling of LFM near NIP (Fig. S3a, c). Block artifacts simultaneously disturb structure continuity in both lateral and axial axes (Fig. S3d, e). The third kind of image artifact is the defocus artifact among different layers. We find a sphere at *z* = −70 µm shows smear ghost with high-frequency grids and blocks even at *z* = +50 µm after LFM reconstruction, which is very different from a conventional widefield microscope whose defocus pattern is smooth (Fig. S4a). When there are two spheres in the space, the original well-reconstructed sphere can be contaminated by grid patterns due to the defocus of other spheres (Fig. S9). When imaging a thick biological volume with LFM, each depth layer generates a *z*-spread defocus pattern that contains high-frequency components mixing with other depths (Figs. S4b, f–h). The resulting reconstructions will be full of gird-like patterns and have isolated unnatural high-frequency components in the Fourier domain (Fig. S4c).

To address all of the artifacts simultaneously, we propose DiLFM, which includes a few RL iterations to avoid edge ringing and an additional dictionary patching approach to suppress other artifacts and improve the imaging contrast. The number of RL iterations in this approach is carefully chosen such that beyond that number the ringing artifact appears (Fig. S2a, b, Table S2). Our DiLFM leverages the domain similarity of different biological samples to assist high-fidelity recovery in LFM (Methods). Compared to traditional LFM, DiLFM achieves the same contrast as RL deconvolutions with 10 iterations but with significantly reduced edge ringing (Fig. S2f). Meanwhile, DiLFM faithfully recovers the block-like feature of a round bead in both *x–y* and *x–z* planes compared to traditional LFM (Fig. S3b–d). Compared to frequency-domain filtering methods, DiLFM can achieve higher contrast over different depths (Fig. S7). The grid-like crosstalk artifacts by traditional LFM are suppressed in DiLFM and reconstructed samples become much smoother (Fig. S4d, i–k, Fig. S9b). Such artifact reduction is also confirmed in the frequency domain since the reconstruction by DiLFM has a more natural and clearer frequency response (Fig. S4e) compared to that by traditional LFM.

We further compare our DiLFM with other emerging LFM methods^28,29^ through simulation. We see all methods apart from RL deconvolution from traditional LFM suppress the block-like artifacts, while anti-aliasing filter blurs the sample, and phase space method generates ringing artifacts (Fig. 3a–c). DiLFM achieves the artifact-suppressed reconstruction together with a sharp edge which shows a great balance between artifact reduction and resolution maintenance. We use an imaging quality metric called structural similarity index (SSIM)^31^ to quantitatively assess the reconstruction quality. We find the SSIM by our method is 0.89, which is much higher than 0.81 of phase space approach, 0.69 of RL with an anti-aliasing filter, and 0.65 of RL deconvolution. For objects with gradual changes in the intensity profiles, our DiLFM also achieves superior reconstruction results compared to other methods (Fig. S8).

**Fig. 2:**
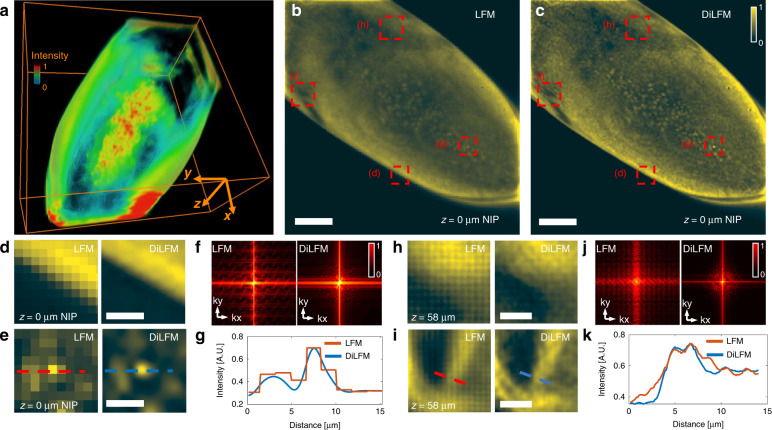
DiLFM suppresses artifacts and increases the contrast in reconstructing the Drosophila embryo. **a** Reconstructed Drosophila embryo in a depth range of 164 µm. **b**, **c** Reconstructed *z* = 0 µm layer (NIP) by RL and DiLFM, respectively. **d**, **e** Zoom-in area marked by the red dashed boxes in (**b**) at *z* = 0 µm (NIP) by RL (left) and DiLFM (right). **f** Fourier transform of (**d**) in the log scale. **g** Intensity profile along the dashed line in (**e**) by RL (blue) and DiLFM (red). **h**, **i** Zoom-in areas marked by red dashed boxes in (**b**) at *z* = 58 µm by RL (left) and DiLFM (right). **j** Fourier transform of (**h**) in the log scale. **k** Intensity profiles along the dashed line in (**i**) by RL (red) and DiLFM (blue). Scale bars in (**b**) and (**c**) are 50 µm, in (**d**), (**e**), (**h**), (**i**) are 10 µm

### DiLFM improves fidelity in biological observations

The artifact reduction makes LFM more reliable in observing *Drosophila* embryos and adult *Drosophila* brains (Fig. 2a and Fig. S5a). We observe that the block-wise artifacts are largely reduced by DiLFM, and the embryo boundary and brain sulcus are restored to be smooth (Fig. 2d, f, Fig. S5i–k). Embryo cells at NIP that are incorrectly reconstructed into square forms by traditional LFM are restored to be round by DiLFM (Fig. 2e, g). The frequency response of these structures is restored to be a natural form with reduced periodic artifacts (Fig. 2f and Fig. S5k). We also observe the grid patterns from depth crosstalk are largely reduced by DiLFM (Fig. 2h, i, Fig. S5f–h). We find the peak-to-valley ratio of a brain sulcus is improved ~1.2 times at *z* = 10 µm by DiLFM, underlining the reduction of artifacts in DiLFM does not sacrifice resolution, as compared to previous methods.

**Fig. 3:**
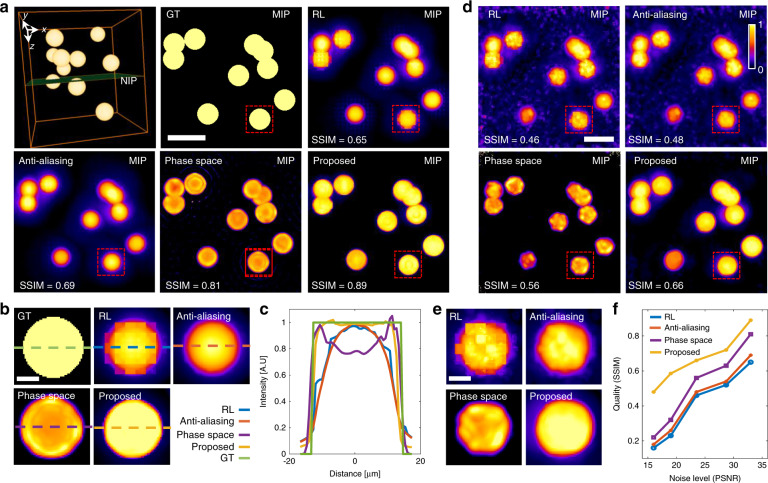
Reconstruction quality comparisons among different light field reconstruction methods numerically. **a** 3D rendering of the volume with spherical objects and the maximum intensity projections (MIP) of ground truth volume (GT) and reconstructed volumes by RL, RL with an anti-aliasing filter, phase space, and DiLFM. Structural similarity index (SSIM) of different approaches are labeled in each image. **b** Zoom-in panels of one of the spherical objects indicated by the red dashed boxes in (**a**), which shows DiLFM achieves sharp edge and uniform intensity inside the sphere. **c** Intensity profile along the dashed line in (**b**). **d** MIP of reconstructions by different approaches under mixed Gaussian and Poisson noise (noise level PSNR = 23.5). **e** Zoom-in panels of one of the spherical objects indicated by the red dashed boxes, which shows DiLFM achieves the least-distorted result. **f** SSIM of different reconstructions in noise levels from PSNR = 15.8 to 33.2 dB. Scale bars in (**a**) and (**d**) are 50 µm, in (**b**) and (**e**) are 10 µm

LFM is widely used in high-speed volumetric recording tasks due to its scanning-free and low phototoxicity, compared to scanning techniques such as confocal microscopy and two-photon microscopy. We thus demonstrate the superior performances of DiLFM in zebrafish blood flow imaging in vivo at 100 Hz in 3D (Fig. S6). We find DiLFM achieves reduced background compared to traditional LFM (Fig. S6b, c). Blood cells are with reduced depth crosstalk in DiLFM such that they can be easily tracked (Fig. S6d, e). High-speed volumetric recording enables us to analyze blood flows in zebrafish larvae by calculating time-lapse intensity changes through a blood vessel cross-section. Such intensity fluctuations fail to predict blood cell flows in traditional LFM due to low contrast and artifacts (Fig. S6f, h). In the contrast, DiLFM provides clear reconstructions and suppresses the ambiguity in blood cell counting (Fig. S6g, i, Fig. S11).

### DiLFM achieves significant noise robustness

As a volumetric imaging tool, LFM shows much lower phototoxicity compared to a confocal microscope since almost all emitted fluorescent photons contribute to the final image without waste. However, the illness of LFM reconstruction causes severe artifacts in low photon flux conditions. The dominant source of noise in LFM is the shot noise, which can be modeled as a Poisson distribution^13^, while the readout noise following Gaussian distribution also contaminates the image. Although traditional LFM reconstruction is derived through Poisson noise, its performance drops quickly when noise is severe and other types of noise appear. Our DiLFM can intake mixed Poisson and Gaussian noise contamination as a prior during the training process (Methods), and prevent noise-induced artifacts and resolution degradations. We show DiLFM achieves superior performance under different noise levels compared to other methods in numerical simulations (Fig. 3d–f). When the noise level is at 23.5 dB peak signal-to-noise ratio (PSNR), we find the DiLFM has clear background while RL and anti-aliasing methods still have noisy pixels remained (Fig. 3d). DiLFM achieves the least distorted reconstructions of simulated spheres among all methods with the highest SSIM (Fig. 3e). We conduct reconstruction quality assessment through different noise levels (PSNR ranges from 15.9 dB to 33.2 dB) and find DiLFM achieves the best reconstruction quality across the whole noise range (Fig. 3f). Especially, when PSNR drops below 20 dB, all other methods show a significant performance drop while our method remains high performance. Similar performance improvement by DiLFM is also observed in samples with gradual changes in intensity profiles (Fig. S8).

**Fig. 4:**
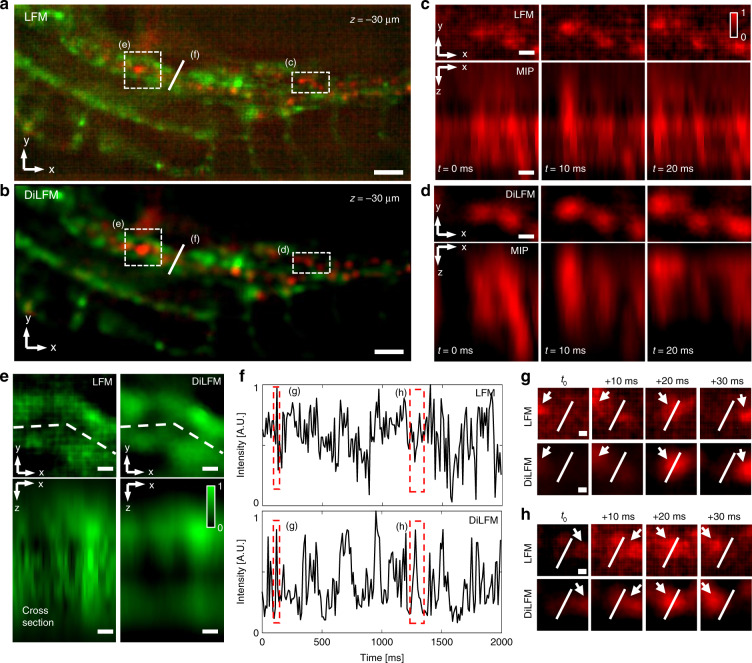
High-speed Zebrafish blood flow imaging in 3D under extremely low illumination power. **a**, **b** Reconstructed zebrafish blood cell volumes by LFM and the DiLFM at *z* = −30 µm under 0.15 mW mm^−2^ (488 nm) and 0.12 mW mm^−^^2^ (561 nm) illumination, respectively. The red color shows the blood cells and the green color shows blood vessel walls. **c** Zoom-in area marked by the white dashed box in (**a**) and corresponding maximum intensity projection (MIP) along the *y*-axis by RL at different time stamps. **d** Zoom-in area marked by the white dashed box in (**b**) and corresponding MIP along the *y*-axis by DiLFM at different time stamps. **e** Zoom-in area marked by the white dashed box in (**a**) by LFM (left column) and in (**b**) by DiLFM (right column). **f** Time-lapse reconstructed intensity along the white line in (**a**) by LFM (top) and by DiLFM (bottom) at *z* = −30 µm. **g**, **h** Zoom-in area around the white line in (**a**) and (**b**) in 30 ms time window by LFM (top) and DiLFM (bottom). The time window is indicated by the dashed box in (**f**). Arrows mark the blood cell at *z* = −30 µm at different time stamps. The white lines in (**g**) and (**h**) are the same as the lines in (**a**) and (**b**). Scale bars in (**a**) and (**b**) are 50 µm, in (**c**–**e**) are 10 µm, in (**g**) and (**h**) are 5 µm

The robust performance of DiLFM in noisy conditions fully unleashes the potential of LFM in long-term in vivo observation of living zebrafish, where illumination heat damage needs to be carefully avoided. To confirm such potentials, we image the zebrafish blood cells (erythrocytes) with only 0.12 ${\mathrm{mWmm}}^{ - 2}$ laser power compared to 6.8 ${\mathrm{mWmm}}^{ - 2}$ used in previous experiments (Fig. S6) while the imaging rate remains at 100 Hz. We find the traditional LFM reconstruction at *z* = -30 µm is noisy with unrecognizable blood vessels and cells due to extremely low laser power (Fig. 4a, c, e). On the other hand, DiLFM restores clear structures with reduced background (Fig. 4b, d, e). The quality improvement by DiLFM is 3D instead of 2D, confirmed through better resolved hollow-core vessels and elliptical blood cells (Fig. 4d, e). Benefitting from the improved image quality, we show the DiLFM significantly increases the counting accuracy of flowing blood cells by simply calculating cross-section intensity fluctuations (Fig. 4f, h), compared to traditional LFM which has a highly noisy baseline and is hard to judge cell flows (Fig. 4f, g). Blood cells reconstructed by DiLFM have much more compact and clearer profiles, which reduces ambiguity in cell counting (Fig. S10).

**Fig. 5:**
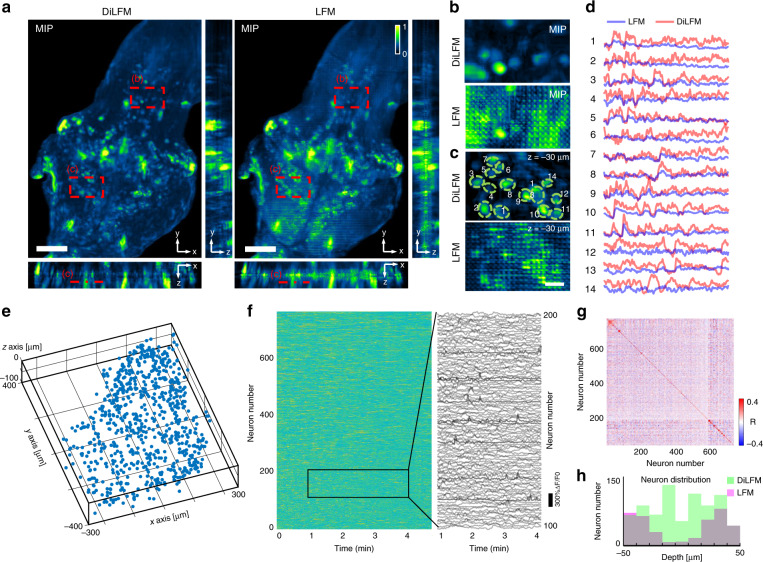
Zebrafish calcium imaging in vivo. **a** Maximum intensity projection (MIP) along *x*, *y*, and *z* axes of temporal summarized volume (Methods) by DiLFM (left) and traditional LFM (right). The summarized volume is the standard deviation of each reconstructed volume in the time domain. **b** Zoom-in area marked by red dashed box in (**a**). **c** Zoom-in area marked by red dashed box in (**a**) with manually labeled neurons shown as green circles. **d** Inferred neuron activities through CNMF-E of manually labeled neurons in (**c**) by DiLFM (red) and traditional LFM (blue). **e** 3D distribution of inferred neurons by DiLFM. **f** Left, neuron activities of 779 DiLFM inferred neurons. Right, zoom-in activities of 100 neurons in a 3 min recording window. **g** Correlation matrix of DiLFM inferred neurons. **h** Neuron distributions along the *z*-axis by DiLFM (green) and traditional LFM (magenta). Scale bars in (**a**) is 100 µm, in (**b**) and (**c**) are 15 µm

Finally, we demonstrate DiLFM can achieve better neuron activity inference compared to the traditional LFM method in low-illumination conditions. We record a HUC: H2B-GCaMP6s larvae zebrafish embedded in 1% agar with 0.37 mW mm^−2^. We visualize the neuron extraction by plotting the projected standard deviation volume along the temporal axis in Fig. 5a (Methods). The thick larvae zebrafish head and low illumination power blur the reconstruction by traditional LFM and generate high backgrounds and artifacts, which severely disturb neuron inference (Fig. 5a). On the other hand, our proposed DiLFM technique obtains sharper images with finer spatial details thanks to the learned dictionary prior. Detectable artifacts due to noise and low spatial sampling of LFM which covers neurons are absent in DiLFM. We find neurons can be much easier to be recognized through DiLFM compared to traditional LFM (Fig. 5c). In the temporal domain, traditional LFM only achieves neuron activities with poor Δ*F*/*F* since the SNR is low, while DiLFM achieves higher activity contrast since the background is largely suppressed and noise is smoothed through the dictionary patching (Fig. 5d). In our experiment, DiLFM unveils 779 neurons through CNMF-E^32^ analysis in a range of 800 $\times$ 600 $\times$ 100 µm^3^ volume, compared to 383 neurons by LFM. The spatial distributions of those active neurons, temporal activities, and temporal correlations are plotted in Fig. 5e-g. The artifacts of traditional LFM prevent CNMF-E from finding neurons near NIP, while neurons found by CNMF-E under the same parameters are uniform along different depths by DiLFM (Fig. 5h). Higher fidelity of inferring neurons in both spatial and temporal domain of DiLFM makes it superior in volumetric functional imaging (Fig. S12). Compared to other LFM techniques which require scanning^33^ or multiview imaging^22^, DiLFM gives an efficient performance-improving solution without any hardware modifications.

## Discussion

In summary, we have developed DiLFM, an algorithm-enhanced LFM technique that can substantially reduce reconstruction artifacts and maintain high contrast without any hardware modification even in extremely noisy conditions. To optimize the performance of the proposed DiLFM, we thoroughly discuss the appearance and mechanism of three different kinds of LFM reconstruction artifacts and intake them all into a dictionary patching model to correct them. Furthermore, the proposed dictionary patching increases the reconstruction resolution and contrast by supplying high-resolution and high-contrast information from the training stage. We validate our DiLFM through both simulations and experiments, including imaging *Drosophila* embryos and *Drosophila* brain. We further show DiLFM can increase the cell-counting accuracy of flowing blood cells in zebrafish in vivo even under extremely noisy conditions to keep the animal safe in long-term recordings. In the functional imaging experiment, we show DiLFM can discover two times more neurons with improved Δ*F*/*F* and reduced artifacts disturbance with low light dosage. We hope the scheme can help LFM become a promising and reliable tool for high-speed imaging biological tissues in 3D.

The proposed DiLFM achieves superior performance compared to traditional LFM but with full advantages of LFM in other aspects. For example, the volume acquisition rate of DiLFM is independent of the size of the sample and only limited by camera frame rate, compared to other 3D imaging technology. Introducing the dictionary only affects the downstream data processing speed without any sacrifice of capturing rate. It is straightforward to extend DiLFM to a larger FOV or a compact head-mounted LFM^27^. Furthermore, by introducing photon-scattering models into dictionary priors^34^, it is possible to exceed the depth-penetration limitation of DiLFM in in vivo mouse-brain imaging^23^. Borrowing the thoughts from DiLFM of using the versatile dictionary to adopt different imaging environments, other deconvolution energized volumetric imaging methods^22,35^ can also use such a prior for better performance in various applications.

## Materials and methods

### DiLFM optical setup

We set up the light field microscope based on a commercial microscope (Zeiss, Observer Z1) and use a mercury lamp as the illumination source. We use different objectives for different imaging tasks (see Table S1) with the same $f$ = 165 mm tube lens. The MLA is put on the image plane of the microscope. The specification of the MLA we use has a 100 µm pitch size and a 2.1 mm focal length to code the 3D information. We put a relay system between the camera (Andor Zyla 4.2 Plus, Oxford Instruments) and the MLA, which conjugates the back-pupil plane of the MLA to the sensor plane. The sensor pixel size is 6.5 µm and the magnification of the relay lens system is set to be 0.845.

### DiLFM principle

The proposed DiLFM can be decomposed into two parts: raw reconstructions through a few runs of RL iterations and fine reconstructions through dictionary patching. In the following sections, we first mathematically represent the RL iteration of LFM, then describe the way that our proposed dictionary patching fixes these artifacts and improves the reconstruction resolution and contrast.

### Light-field microscopy model and RL deconvolution

A common light-field microscope is composited by a wide-field microscopy and an MLA put in the native imaging plane, as shown in Fig. 1. We denote the sample space coordinate as $\left( {x_1,x_2,z} \right)$ and sensor space coordinate as $\left( {s_1,s_2} \right)$. The point spread function (PSF) of LFM can be formulated by

1 $$\begin{array}{*{20}{c}} {h\left( {x_1,x_2,z,s_1,s_2} \right) = \left| {\Im _{f_\mu }\left\{ {U\left( {x_1,x_2,z,s_1,s_2} \right){\Phi}\left( {s_1,s_2} \right)} \right\}} \right|^2} \end{array}$$

Here, $U\left( {x_1,x_2,z,s_1,s_2} \right)$ is the optical field in the NIP generated by a point source in $\left( {x_1,x_2,z} \right)$, which is defined by^36^

2 $$\begin{array}{l}U\left( {x_1,x_2,z,s_1,s_2} \right)\\\;\;\; = \frac{M}{{f_{obj}^2\lambda ^2}}\exp\left({ - \frac{{iu}}{{4\sin ^2\left({\alpha /2} \right)}}} \right)\mathop {\int}\nolimits_0^\alpha P\left(\theta \right)\exp\left({ - \frac{{iu\sin ^2\left({\theta /2} \right)}}{{2\sin ^2\left({\alpha /2} \right)}}} \right)J_0\left({\frac{{\sin \left(\theta \right)}}{{\sin \left(\alpha \right)}}v} \right)\sin \left(\theta \right){\mathrm{d}}\theta \\v \approx k\sqrt {\left({x_1 - s_1} \right)^2 + \left( {x_2 - s_2} \right)^2} \sin \left(\alpha \right) \\{u \approx 4kz\sin ^2\left({\alpha /2} \right)} \end{array}$$

${\Phi}\left( {s_1,s_2} \right)$ is the modulation function of the MLA which has pitch size $d$ and focal length $f_\mu$

3 $$\begin{array}{l}{\Phi}\left( {s_1,s_2} \right) = {\iint} {\mathrm{rect}}\left( {\frac{{t_1}}{d}} \right){\mathrm{rect}}\left( {\frac{{t_2}}{d}} \right)\exp \left( { - \frac{{ik}}{{2f_\mu }}\left( {t_1^2 + t_2^2} \right)} \right)\\comb\left( {\frac{{s_1 - t_1}}{d}} \right)comb\left( {\frac{{s_2 - t_2}}{d}} \right) dt_1dt_2\end{array}$$

$\Im _{f_\mu }\left\{ \cdot \right\}$ is the Fresnel propagation operator which carries a light field as input and propagates a distance $f_\mu$ along the optical axis.

To reconstruct the 3D sample from the captured image, we need to bin the continuous sample and sensor space for voxelization and pixelization^13^. LFM can then be modeled as a linear system $H$ that maps the 3D sample space into 2D sensor space

4 $$\mathop {\sum}\limits_{x_1,x_2,z} {H\begin{array}{*{20}{c}} {\left( {x_1,x_2,z,s_1,s_2} \right)X\left( {x_1,x_2,z} \right) = Y\left( {s_1,s_2} \right)} \end{array}}$$

Here *Y* is the discrete sensor image and *X* is the 3D distribution of the sample. The weight matrix *H* can be sampled from Eq. (1) which records how the photons emitted from the voxel $(x_1,x_2,z)$ separates and contributes to the pixel $(s_1,s_2)$. Further, the weight matrix $H$ could be simplified via periodicity introduced by the MLA, which implies

5 $$\begin{array}{*{20}{c}} {H\left( {x_1,x_2,z,s_1,s_2} \right) = H\left( {x_1 + D,x_2 + D,z,s_1 + D,s_2 + D} \right)} \end{array}$$

where $D$ is the pitch of microlens under the unit of pixel size. We simplify Eq. (4) into ${\mathbf{H}}_{{\mathrm{for}}}\left( X \right) = Y$ to represent the forward projection in LFM. On the other hand, if we trace back each light ray that reaches the sensor, we can rebuild the sample $X(x_1,x_2,z)$ via

6 $$\mathop {\sum}\limits_{s_1,s_2}{\frac{{ {H\left( {x_1,x_2,z,s_1,s_2} \right)Y\left( {s_1,s_2} \right)} }}{{\mathop {\sum}\nolimits_{w_1,w_2} {H\left( {x_1,x_2,z,w_1,w_2} \right)} }} = X\left( {x_1,x_2,z} \right)}$$

We simplify Eq. (6) into ${\mathbf{H}}_{{\mathrm{back}}}\left( Y \right) = X$ to represent the backward projection in LFM. It is popular to use RL algorithm to refine *X* from *Y* and *H*. In each iteration, RL tries to update $\hat X^{\left( t \right)}$ from the last iteration result $\hat X^{\left( {t - 1} \right)}$ via^13^

7 $$\begin{array}{*{20}{c}} {\hat X^{\left( t \right)} \leftarrow \hat X^{\left( {t - 1} \right)} \odot {\mathbf{H}}_{{\mathrm{back}}}\left( {\frac{Y}{{{\mathbf{H}}_{{\mathrm{for}}}\left( {\hat X^{\left( {t - 1} \right)}} \right)}}} \right)} \end{array}$$

where $\odot$ means element-wise multiplication. We denote the running Eq. (7) once as one RL iteration. Usually to reconstruct an LFM volume requires multiple RL iterations^37^. On the other hand, running RL iterations too much will cause severe edge ringing problems.

### Fixing artifacts and improve contrast through dictionary patching

In this section, we first show how to learn a dual dictionary pair $\left( {{\mathbf{D}}_{l,z},{\mathbf{D}}_{h,z}} \right)$ with LFM model, where ${\mathbf{D}}_{l,z}$ is the collection of most representative elements of raw LFM reconstruction and ${\mathbf{D}}_{h,z}$ is the collection of corresponding high-fidelity and artifact-reduced elements. The element here means the local features of an image, e.g., corners for edges. We then show how to apply the learned dictionaries to achieve high-fidelity and artifact-reduced reconstruction from raw RL reconstruction.

We prepare a set of high-fidelity and high SNR 3D volume $\left\{ {I_j^{ref}} \right\}$ to learn the dictionary prior. For each reference volume $I_j^{ref}$, we numerically feed it into LFM forward projection built-in Eq. (4) to get an LFM capture $Y_j^{ref}$, then use the RL deconvolution in Eq. (7) to get a raw reconstructed volume $\hat I_j^{ref}$. In this way, we generate a set of high and low-fidelity volume pairs $\left\{ {\left( {I_j^{ref},\hat I_j^{ref}} \right)} \right\}$, where the resolution drops and artifacts in $\hat I_j^{ref}$ are generated through the real LFM model. Since the LFM artifacts are associated with depth z as discussed in Sec. 2.2, we split the volume pair $\left\{ {\left( {I_j^{ref},\hat I_j^{ref}} \right)} \right\}$ into different *z*-depth pairs $\left\{ {\left( {I_{j,z}^{ref},\hat I_{j,z}^{ref}} \right)} \right\}$ and further generate a patch dataset ${\cal{P}}_z$ regarding *z*-depth for the following training via

8 $$\begin{array}{*{20}{c}} {{\cal{P}}_z = \left\{ {L_k\left( {I_{j,z}^{ref} - \hat I_{j,z}^{ref}} \right),L_k\left( {F\hat I_{j,z}^{ref}} \right)} \right\} \buildrel \Delta \over = \left\{ {p_h^k,p_l^k} \right\}} \end{array}$$

where $L_k\left( \cdot \right)$ is the linear image-to-patch mapping so that a $\sqrt n \times \sqrt n$-pixel patch can be extracted from an image and $k$ is the patch index. Patches are randomly selected from the image with overlapping. Since some biological samples are quite sparse, we select patches with enough signal intensity to avoid null patches. $F$ is a feature extraction operator that provides a perceptually meaningful representation of patch^38^. The common option of $F$ can be the first- and second-order gradients of patches. The reason to use $I_{j,z}^{ref} - \hat I_{j,z}^{ref}$ is to let the later learning process focus on high-frequency information^30^. We also conduct a dimensionality reduction through Principal Component Analysis (PCA) algorithm to $\left\{ {p_l^k} \right\}$ for reducing superfluous computations^30^. After these preparations, the low-fidelity dictionary ${\mathbf{D}}_{l,z}$ which is the collection of most representative elements in $z$th depth of LFM reconstructed biological tissue can be learned via

9 $$\begin{array}{l} {{\mathbf{D}}_{l,z},\left\{{\beta ^k} \right\} = {\mathrm{argmin}}\displaystyle\mathop {\sum}\limits_k \Vert p_l^k - {\mathbf{D}}_{l,z}\beta ^{k}\Vert_{2}^{2},{s.t.}\Vert\beta ^{k}\Vert_0 \,\le\, \kappa ,\forall k} \end{array}$$

where $\Vert\cdot \Vert_2$ is $\ell _2$ norm which measures the data fidelity, $\Vert\cdot \Vert_0$ is the $\ell _0$ “norm” which measures the sparsity, $\beta _k$ is the sparse representation coefficients for low-fidelity patch $p_l^k$, and $\kappa$ is maximum sparsity tolerance. Equation (9) could be effectively solved by the well-known K-SVD algorithm^39^. The corresponding high-fidelity dictionary ${\mathbf{D}}_{h,z}$ is generated by solving the following quadratic programming (QP)

10 $$\begin{array}{*{20}{c}} {{\mathbf{D}}_{h,z} = {\mathrm{argmin}}\mathop {\sum}\limits_j \left\Vert{I_{j,z}^{ref} - \hat I_{j,z}^{ref}} - \left[ {\mathop {\sum}\limits_k {L_k^TL_k} } \right]^{ - 1}\left[ {\mathop {\sum}\limits_k {L_k^T{\mathbf{D}}_{h,z}\beta ^k} } \right]\right\Vert_2^2} \end{array}$$

Note the library pair $\left( {{\mathbf{D}}_{l,z},{\mathbf{D}}_{h,z}} \right)$ is specific for different $z$ since the degradation of imaging quality is depth-dependent. Here we assume the high- and low-fidelity dictionaries share the same sparse representation $\left\{ {\beta ^k} \right\}$ based on the assumption that artifact contamination and blur operation in LFM reconstructions are near-linear (Note S1). The NIP artifact is covered by the dictionary learned in the NIP layer. The defocus artifact is also covered since the whole reconstructed volume is learned instead of only learning single-image reconstructions, as a comparison to the traditional dictionary learning method^38^. The high-fidelity and artifact-free reference volume $\left\{ {I_j^{ref}} \right\}$ are collected from broad bioimage benchmark collection Nos. 021, 027, 032, 033^40^, and SOCR 3D Cell Morphometry Project Data^41^. The flowchart of the LFM dictionary learning process is shown in Fig. S1a.

To achieve high-fidelity and artifact-reduced volume $\tilde X^{(t)}$ from raw RL reconstruction volume $\hat X^{(t)}$, we run sparse representation for each z depth of $\hat X^{(t)}$ with the learned *z*-depth dictionary prior $\left( {{\mathbf{D}}_{l,z},{\mathbf{D}}_{h,z}} \right)$. Firstly, we estimate the sparse representation of each local patch of $\hat X_z^{(t)}$. We extract the local patch from $\hat X_z^{(t)}$ by the same mapping $L_k\left( \cdot \right)$ as above with the size of $\sqrt n \times \sqrt n$-pixel, then search a sparse coding vector $\alpha _z^k$ such that $L_k\hat X_z^{(t)}$ can be sparsely represented as the weighted summation of a few elements from ${\mathbf{D}}_{l,z}$

11 $$\begin{array}{*{20}{c}} {\min\!\Vert \alpha_{z}^k\Vert_0,\qquad {s.t.}\Big\Vert FL_k\hat X_z^{(t)} - {\mathbf{D}}_{l,z}\alpha_{z}^k \Big\Vert_2\, \le\, \in} \end{array}$$

where ${\it{\epsilon }}$ is the error tolerance. Eq. (11) can be solved via orthogonal matching pursuit (OMP) algorithm^42^. Secondly, we use the found sparse coefficients $\alpha _z^k$ to recover the high-fidelity and artifact-reduced patch $p_{h,z}^k$ by $p_{h,z}^k = {\mathbf{D}}_{h,z}\alpha ^k$, then accumulate $p_{h,z}^k$ to form a high-fidelity image $\tilde X_z^{(t)}$ by solving the following minimization problem

12 $$\begin{array}{*{20}{c}} {\tilde X_z^{(t)} = {\mathrm{argmin}}\displaystyle\mathop {\sum}\limits_k \Big\Vert{L_k} \left({\tilde X_z^{(t)} - \hat X^{\left(t \right)}} \right) - p_{h}^{k}} \Big\Vert_2^2 \end{array}$$

After concatenating $\tilde X_z^{(t)}$ into the whole volume $\tilde X^{(t)}$, a high-fidelity and artifact-reduced volume is recovered from original RL reconstruction $\hat X^{(t)}$. The flow-chart of the reconstruction processing is shown in Fig. S1b. To choose proper RL iterations before dictionary patching, one can visually check the RL output. Once there is edge ringing the RL iteration number should be reduced. For samples with uniform intensity distribution, 1 RL iteration is enough. All RL iteration numbers of experiments in the manuscript can be found in Table S2.

### Dictionary training with noise

We train the dictionary with mixed Poisson and Gaussian noise contaminations. The dark noise and the photon noise of fluorescent imaging follow a Poisson distribution while the readout noise follows a Gaussian distribution. Hence, we choose the mixed Poisson and Gaussian noise to mimic the real situation. The observed image under the microscope thus can be modeled as^43^

13 $$\begin{array}{*{20}{c}} {Y = \alpha {\rm{P}}\left( {\frac{{{ \mathbf{H} }_{{\mathrm{for}}}\left( X \right)}}{\alpha }} \right) + {\mathbb{N}}\left( {0,\sigma ^2} \right)} \end{array}$$

where $Y$ is observed image, ${\mathbf{H}}_{{\mathrm{for}}}$ is the forward propagator of LFM, $X$ is the noise-free sample, $\alpha$ is the scaling factor that controls the strength of Poisson noise, ${\mathrm{P}}( \cdot )$ is the realization of Poisson noise, and ${\mathbb{N}}\left( {0,\sigma ^2} \right)$ represents Gaussian noise with 0 mean and $\sigma ^2$ variance. We fix $\sigma ^2$ to be ~200 for 16-bit sCMOS image, and varying $\alpha$ to generate captures with the different noise levels. The high-fidelity and artifact-free reference volume $\left\{ {I_j^{ref}} \right\}$ are firstly propagated to the sensor plane, then added Poisson and Gaussian noise with MATLAB function imnoise to form $\left\{ {\hat I_j^{ref}} \right\}$. Then, noise aware dictionary is learned through Eqs. (9) and (10). $\left\{ {\hat I_j^{ref}} \right\}$ contains multiple levels of noise to accommodate different SNR conditions. Trained low- and high-fidelity dictionaries have different element numbers and patch sizes to accommodate different modalities, see Table S2.

### Sample preparation

#### Drosophila embryo imaging

The *Drosophila* embryo used in this study (Fig. 2) expressed histone tagged with EGFP (w; His2Av::eGFP; Bloomington stock #23560). The embryos were collected by putting adult flies on a grape-juice agar plate for 45 min–1 h. After incubation at 25 °C for 1 h, the embryos were attached to a glass slide with double-sided tape. We use forceps to carefully roll an embryo on the tape until the embryo dechorionated. The Dechorionated embryos were embedded in 2% low-melting-temperature agarose in a Glass Bottom Dish (35 mm Dish with 20 mm Bottom Well, Cellvis). We put the Glass Bottom Dish on the microscope stage and scan the embryo along the *z*-axis 4 times with a 30 µm stride, then concatenate 4 reconstructed stacks to form the volume.

#### Drosophila brain imaging

The *Drosophila* Adult Brain (w1118) used in this study (Fig. S5) was dissected at 4–5 days after eclosion in phosphate buffer saline (PBS) and fixed with 4% paraformaldehyde in PBST (PBS with 0.3%Triton X-100) for 30 min. After washing in PBST, the brain was blocked in 5% normal mouse serum in PBST for 2 h in RT (room temperature) and then immunostained using commercial antibodies. The brain was incubated in primary antibodies (Mouse anti nc82, 1:20, Hybridoma Bank) and secondary antibodies (Goat anti-mouse Alexa-488,1:200, Invitrogen) for 48–72 h at 4 °C, with a 2 h wash at 4 °C between the primary and secondary antibody incubations. After that, the brain was washed 3–4 times in PBST. The brain is cut into ~60 µm thickness slices. The slice was mounted and was further observed by the LFM in epifluorescence mode. No concatenation is made. No further deconvolution is applied.

#### Zebrafish blood cell imaging

Zebrafish from the transgenic line Tg(gata1:DsRed) were used in this study for blood cell imaging (Fig. 4, Fig. S6). For two-color recordings (Fig. 4), zebrafish from the transgenic line Tg(gata1:DsRed) were crossed with zebrafish from the transgenic line Tg(flk: EGFP). The embryos were raised at 28.5 °C until 4 dpf. Larval zebrafish were paralyzed by short immersion in 1$mgml^{ - 1}$ ${\upalpha}$-bungarotoxin solution (Invitrogen). After paralyzed, the larval were embedded in 1% low-melting-temperature agarose in a Glass Bottom Dish (35 mm Dish with 20 mm Bottom Well, Cellvis). We maintained the specimen at room temperature and imaged the zebrafish larval at 100 Hz.

#### Zebrafish functional imaging

Zebrafish from the transgenic line Tg(HUC: GCaMP6s) expressing the calcium indicator GCaMP6s was raised at 28.5°C until 4 dpf for short-term functional imaging (Fig. 1b and Fig. 5). Larval zebrafish were paralyzed by short immersion in 1 mg ml^−1^ ${\upalpha}$-bungarotoxin solution (Invitrogen). After paralyzed, the larval were embedded in 1% low-melting-temperature agarose in a Glass Bottom Dish (35 mm Dish with 20 mm Bottom Well, Cellvis). For imaging, the dorsal side of the head of the larval zebrafish was facing the objective. We maintained the specimen at room temperature and imaged the zebrafish larval at 1 Hz. Assume the reconstructed volume by DiLFM is $\tilde X(x,y,z,t)$ where $\left( {x,y,z} \right)$ is the 3D spatial coordinate of the voxel and $t$ labels the time, the temporal summarized volume was calculated through the following procedures. In the first step, we calculate the rank-1 background components of $\tilde X(x,y,z,t)$ via

14 $$\begin{array}{*{20}{c}} {\left[ {b,f} \right] = \arg \mathop {{\min }}\limits_{b,f} \displaystyle\mathop{\sum}\limits_t \left\Vert{\tilde X(x,y,z,t) - b(x,y,z)} \cdot f\left( t \right)\right\Vert_2^2} \end{array}$$

where $b(x,y,z)$ is the spatial background and $f\left( t \right)$ is the temporal background. $b$ and $f$ can be calculated through normal non-negative matrix factorization techniques^44^. The background-subtracted image is then calculated by $\tilde X_1(x,y,z,t) = \tilde X(x,y,z,t) - b(x,y,z) \cdot f\left( t \right)$. Then, we calculate the standard deviation volume of all the background-subtracted volumes across the time domain via

15 $$\begin{array}{*{20}{c}} {\tilde X_2\left( {x,y,z} \right) = \sqrt {\frac{{\mathop {\sum}\nolimits_t {\left( {\tilde X_1\left( {x,y,z,t} \right) - \frac{{\mathop {\sum}\nolimits_s {\tilde X_1\left( {x,y,z,s} \right)} }}{T}} \right)} ^2}}{T}} } \end{array}$$

where $T$ is the total frame number. In Fig. 5a, we plot the maximum intensity projections of $\tilde X_2\left( {x,y,z} \right)$ along $x$-, $y$-, and $z$-axis to show fired neuron distributions in zebrafish larvae. All captured frames are used for the above calculation.

## Supplementary information

Supplement figures and tables

## Data Availability

The training dataset for *Drosophila* embryo, *Drosophila* brain, zebrafish blood flow, and zebrafish brain imaging experiments are available at https://drive.google.com/drive/folders/1DzYc6NfO1O_jx314Op4ggvV7LT4BTrzB?usp=sharing. Scripts that are used for fluorescent beads simulation can be found in https://github.com/yuanlong-o/DiLFM. The pre-trained dictionary for *Drosophila* embryo imaging and the corresponding DiLFM results are available at https://github.com/yuanlong-o/DiLFM.

## Code availability

Our MATLAB implementation of DiLFM is available at https://github.com/yuanlong-o/DiLFM.
