# Supplementary material for: DiLFM: an artifact-suppressed and noise-robust light-field microscopy through dictionary learning
Source: Light Sci Appl. 2021 Jul 27;10:152. doi: 10.1038/s41377-021-00587-6 (PMC8316327; doi:10.1038/s41377-021-00587-6)
Supplement: Supplementary file 1 — Supplement figures and tables [file 41377_2021_587_MOESM1_ESM.docx]

**Supplementary Information for**

**DiLFM: an artifact-suppressed and noise-robust light-field microscopy through dictionary learning**

**Authors**

Yuanlong Zhang^1,2,3†^, Bo Xiong^1,2,3†^, Yi Zhang^1,2,3^, Zhi Lu^1,2,3^, Jiamin Wu^1,2,3*^, and Qionghai Dai^1,2,3*^

**Affiliations**

^1^ Department of Automation, Tsinghua University, Beijing 100084, China

^2^ Institute for Brain and Cognitive Sciences, Tsinghua University, Beijing 100084, China.

^3^ Beijing National Research Center for Information Science and Technology, Tsinghua University, Beijing 100084, China.

^†^ These authors contribute equally to this work.

^*^ Correspondence and requests for materials should be addressed to [qhdai@mail.tsinghua.edu.cn](mailto:qhdai@mail.tsinghua.edu.cn) (Qionghai Dai) and [wujiamin@tsinghua.edu.cn](mailto:wujiamin@tsinghua.edu.cn) (Jiamin Wu)

**Supplementary Information Table**

| **Figure S1** | Training and reconstruction procedures of DiLFM |
| --- | --- |
| **Figure S2** | DiLFM increases the reconstruction contrast and eliminates edge ringing. |
| **Figure S3** | DiLFM eliminates block-wise artifacts in the native image plane (NIP) and increases axial confinement. |
| **Figure S4** | DiLFM substantially eliminates depth crosstalk artifacts in LFM reconstruction. |
| **Figure S5** | DiLFM eliminates artifacts and increases the contrast in reconstructing *Drosophila* brain slice. |
| **Figure S6** | High-speed Zebrafish blood flow imaging. |
| **Figure S7** | Reconstruction comparisons between LFM, DiLFM and RL+Wiener filter. |
| **Figure S8** | Reconstruction quality comparisons among different algorithms over quadradic intensity spheres ($I=\sqrt{1-r^{2}}$). |
| **Figure S9** | DiLFM reduces depth crosstalk artifacts in dual spheres simulations. |
| **Figure S10** | Blood-cell tracking through Imaris 9.0.1 software in low-power illumination dosage. |
| **Figure S11** | Blood-cell tracking through Imaris 9.0.1 software. |
| **Figure S12** | DiLFM increases neuron segmentation and calcium activities inferring accuracy. |
| **Table S1** | Optical objective and illumination source usages in experiments. |
| **Table S2** | Dictionary training parameters in experiments. |
| **Note S1** | Sparse representation of high- and low-fidelity pairs. |

**Figure S1**


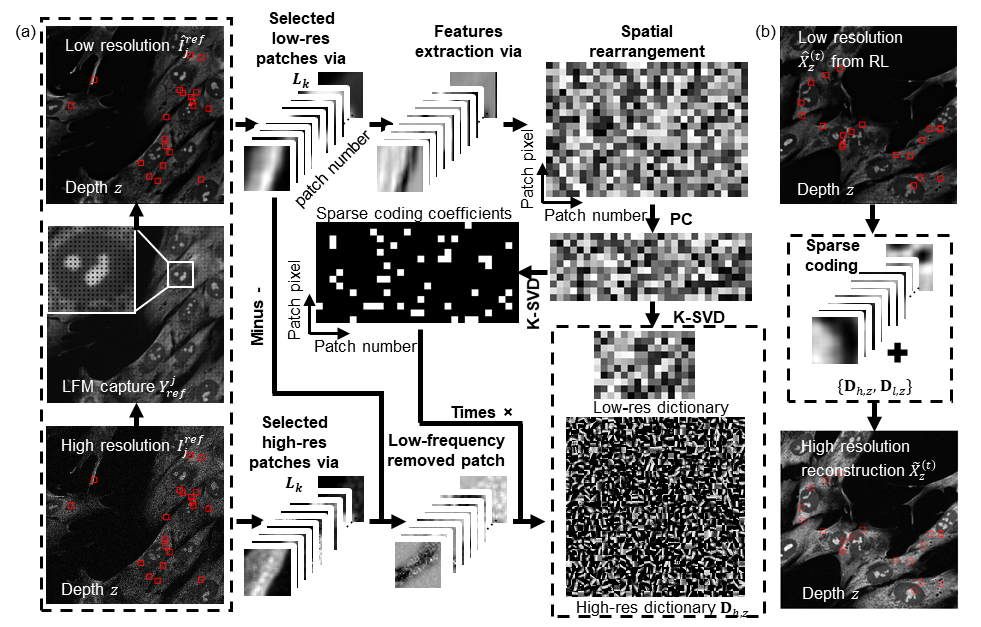


**Fig. S1. Training and reconstruction procedures of DiLFM.**

(a) Dictionary training procedure. A high-fidelity and artifact-free reference volume $I_{j}^{ref}$ is propagated with LFM forward model to the sensor to form a capture $Y_{j}^{ref}$, then $Y_{j}^{ref}$ is reconstructed through RL deconvolution in Eq. (7) to generate low-fidelity and artifact contaminated volume $\hat{I}_{j}^{ref}$. Since the artifacts and resolution drop is *z*-depth specific, we slice both reference volume $I_{j}^{ref}$ and contaminated volume $\hat{I}_{j}^{ref}$ into different layers for the following procedure. The low-fidelity slice $\hat{I}_{j}^{ref}$ is cut into small patches via operator $L_{k}$, and an operator $F$ is used to extract features from each low-fidelity patch. A principal component analysis (PCA) is further used for the patches to reduce superfluous computations. We then use the K-SVD algorithm to learn the sparse representations of the dimension-reduced patches to achieve a low-fidelity dictionary $\boldsymbol{D}_{l,z}$ and corresponding sparse coefficients $\beta^{k}$. On the other hand, we subtract low-fidelity patches from corresponding high-fidelity patches and combine them with $\beta^{k}$ to form a high-fidelity dictionary $\boldsymbol{D}_{h,z}$.

(b) Dictionary reconstruction. With an input low-fidelity reconstructed volume  $\hat{X}^{(t)}$ from RL deconvolution, we slice it into different depth $\hat{X}_{z}^{\left( t \right)}$ and then cut it into small patches through $L_{k}$. We run a sparse coding for each patch based on the low-fidelity dictionary $\boldsymbol{D}_{l,z}$, then use sparse representation coefficients with the high-fidelity dictionary $\mathbf{D}_{h,z}$ to recover the corresponding high-fidelity and artifact-reduced patch. We ensemble all the patches to achieve a high-fidelity image $\tilde{X}_{z}^{(t)}$, and concatenate $\tilde{X}_{z}^{(t)}$ into volume $\tilde{X}^{(t)}$.

**Figure S2**


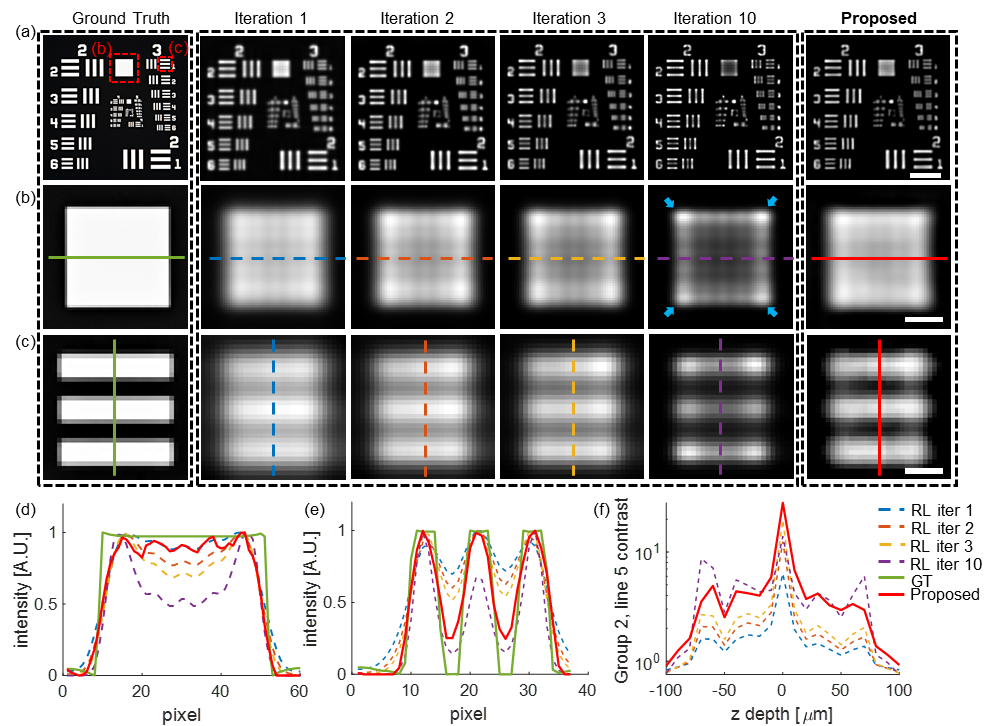


**Fig. S2. DiLFM increases the reconstruction contrast and eliminates edge ringing.**

(a) The ground truth image of a fidelity target at *z*=-50 µm, reconstructions by Richardson-Lucy (RL) deconvolution of traditional LFM with 1, 2, 3, and 10 iterations, and the reconstruction by DiLFM. As the RL iteration goes on, the contrast of the image increases but edge ringing artifacts appear. On the other hand, DiLFM achieves high contrast but with reduced edge ringing.

(b) and (c) are zoomed parts of (a) marked by red dashed boxes, which clearly show DiLFM reduces edge ringing artifacts but simultaneously keeps a high contrast.

(d) Intensity profile of the square structure in (b). We see DiLFM is with minimum edge ringing artifacts, while RL with 10 iterations is mostly distorted.

(e) Intensity profile across group 3, line 1 in (c). We see DiLFM achieves similar contrast as that from RL with 10 iterations.

(f) Contrasts of group2, line 5 across -100 µm to 100 µm depths of different methods. We see DiLFM achieves much better contrast across all the depths over RL with a low number of iterations.

The contrast is calculated to be the peak to valley ratio. Scale bar in (a) is 50 µm, in (b) is 10 µm, in (c) is 5 µm.

**Figure S3**


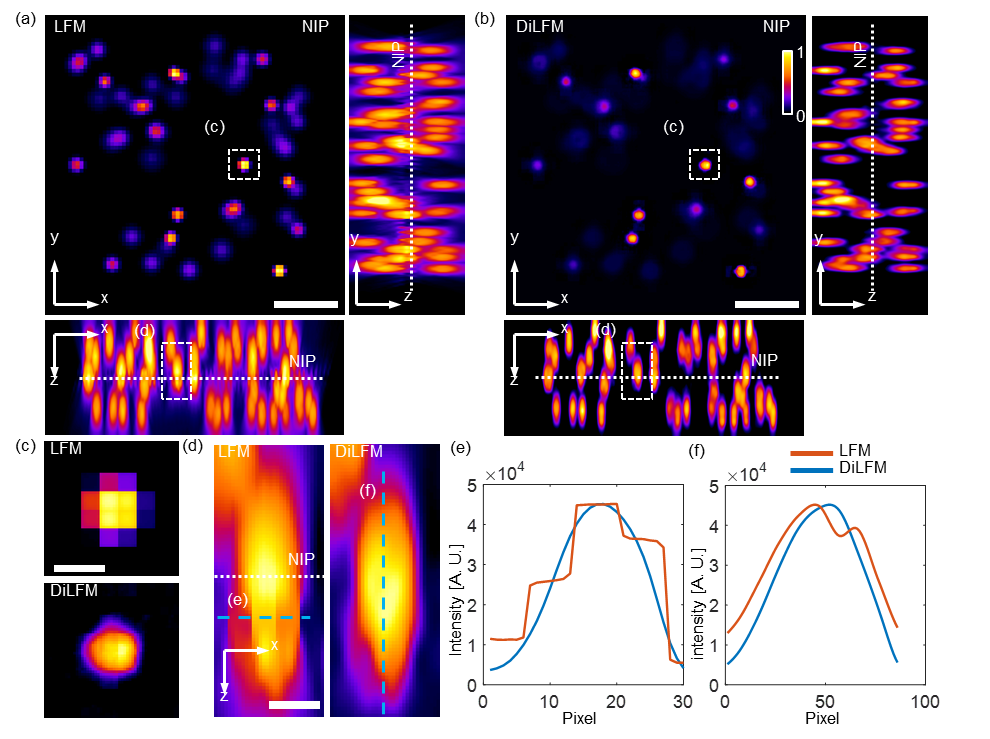


**Fig. S3. DiLFM eliminates block-wise artifacts in the native image plane (NIP) and increases axial confinement.**

(a) and (b) are reconstructions of a 3D sample with randomly distributed beads by traditional LFM and DiLFM, respectively. Here we show the native image plane (NIP) and maximum intensity projections (MIP) along the *y*-axis and *x*-axis. White dotted lines for NIP position.

(c) Zoomed images from NIP images of (a) and (b) marked by white dashed boxes show DiLFM reduces block-wise artifacts.

(d) Zoomed images from *x*-*z* MIP images of (a) and (b) marked by white dashed boxes. White dotted lines for NIP position.

(e) Intensity profile across the horizontal blue dashed line in (d), which shows DiLFM can avoid unnatural step-like wise feature near the NIP.

(f) Intensity profile across the vertical blue dashed line in (d) is with narrower full width at half maximum (FWHM) by DiLFM, showing DiLFM can improve the axial confinement of reconstructed beads.

Scale bars in (a) and (b) are 50 µm, in (c) and (d) are 10 µm.

**Figure S4**


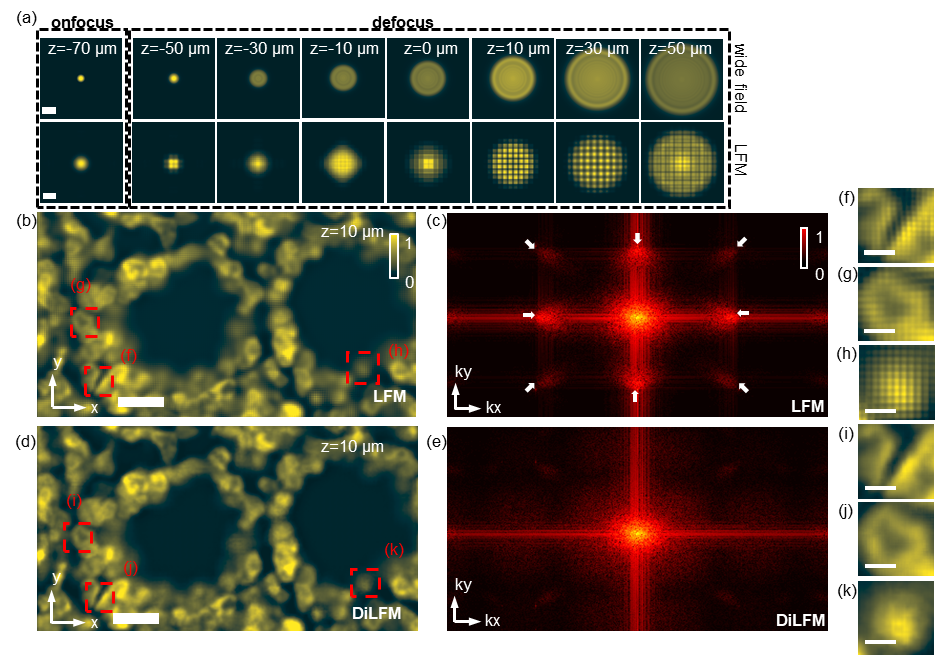


**Fig. S4. DiLFM substantially eliminates depth crosstalk artifacts in LFM reconstruction.**

(a) Reconstructions of a spherical object located in *z*=-70 µm. Even the onfocus reconstruction is clear, other depths in the reconstructed volume will be contaminated by the defocus patterns of the spherical object with high frequency, comparing to the smooth defocus pattern of the wide field.

(b) Reconstructed colon tissue at *z*=10 µm plane by traditional LFM shows low-contrast and grid-like artifacts.

(c) Fourier transformation of the traditional LFM in (c) in the log scale shows several unnatural isolated high-intensity frequency components, which are corresponding to the grid-like artifacts in (b).

(d) The same colon tissue at *z*=10 µm plane but reconstructed by DiLFM, which is high contrast and with reduced grid-like defocus artifacts.

(e) Fourier transformation of DiLFM in (d) in the log scale shows clearer and natural frequency component distributions.

(f)-(h) zoom-in panels from (b) marked by red dashed boxes to show the grid-like defocus artifacts.

(i)-(k) zoom-in panels from (d) marked by red dashed boxes to show the clear structures from DiLFM, as a comparison to (f)-(h).

Scale bars in (a) is 50 µm, in (b) is 100 µm, in (f-h) and (i-k) are 30 µm.

**Figure S5**


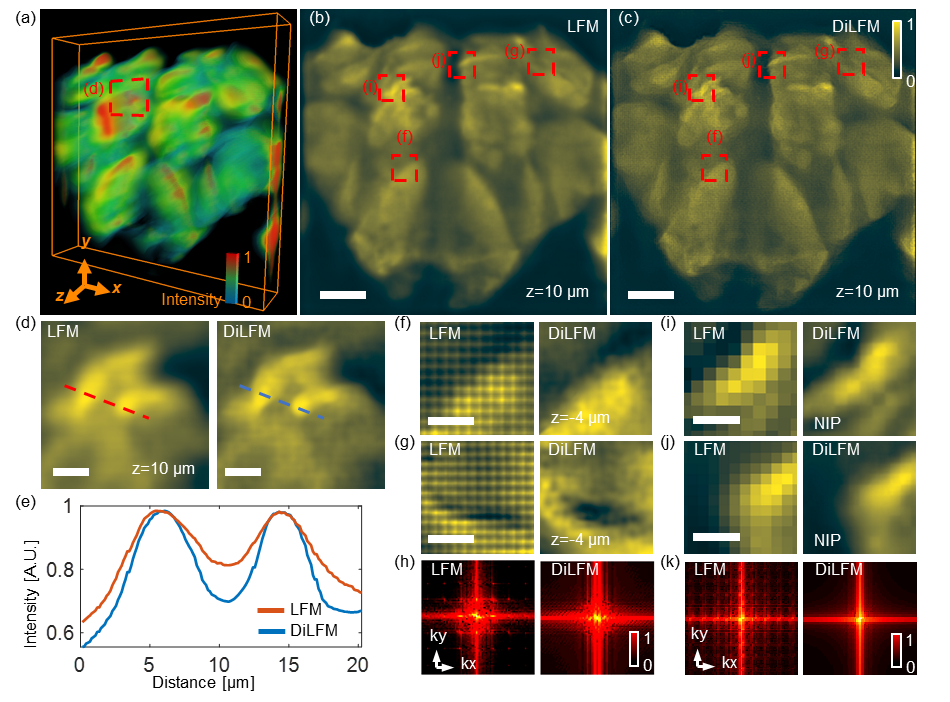


**Fig. S5. DiLFM eliminates artifacts and increases the contrast in reconstructing *Drosophila* brain slice.**

(a) Reconstructed *Drosophila* brain slice rendered in 3D.

(b) and (c) reconstructed *z*=10 µm layer by traditional LFM and DiLFM, respectively.

(d) Zoom-in area marked by red dashed box in (a) at *z*=10 µm by traditional LFM (left) and DiLFM (right).

(e) Intensity profile along the dashed line in (d) by traditional LFM (red) and DiLFM (blue).

(f) and (g), the zoom-in area marked by red dashed box in (b) at *z*=-4 µm by traditional LFM (left) and DiLFM (right).

(h) Fourier transform of (g) in the log scale.

(i) and (j), zoom-in areas marked by red dashed box in (b) at *z*=0 µm (NIP) by traditional LFM (left) and DiLFM (right).

(h) Fourier transform of (j) in the log scale.

Scale bars in (b) and (c) are 50 µm, in (d), (f), (g), (i), and (j) are 10 µm.

**Figure S6**


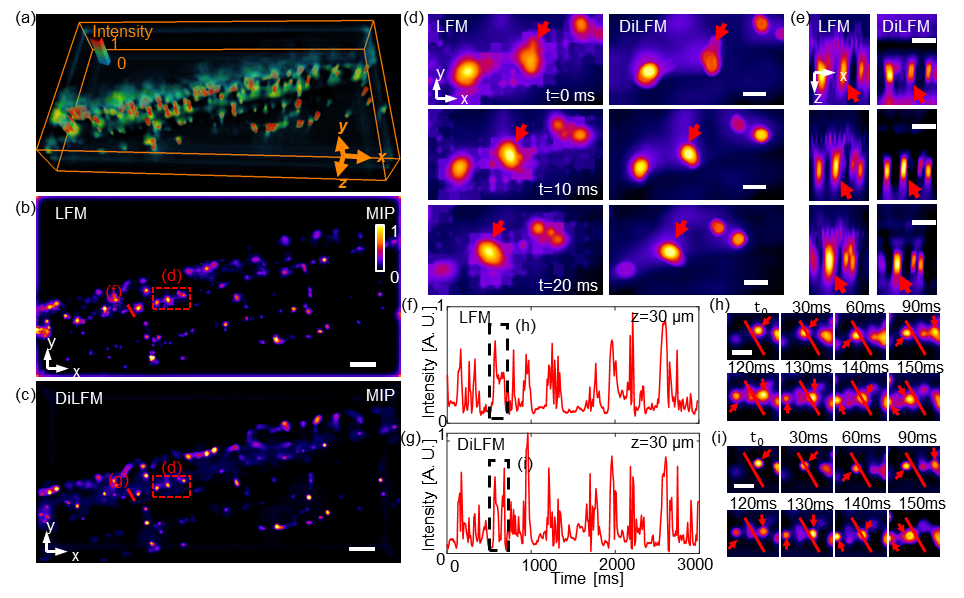


**Fig. S6. High-speed Zebrafish blood flow imaging.**

(a) 3D rendering of DiLFM reconstructed zebrafish blood cells.

(b) and (c) maximum intensity projections (MIPs) of reconstructed zebrafish blood cell volumes by traditional LFM and DiLFM, respectively.

(d) Zoom-in area in (b) by traditional LFM (left column) and in (c) by DiLFM (right column) at t=0 ms, t=10 ms, and t=20 ms. Red arrows mark one of the blood cells at different time stamps.

(e) MIPs along *y*-axis (i.e. *x*-*z* images) of the box regions in (b) by traditional LFM (left column) and in (c) by DiLFM (right column). Red arrows mark the same blood cell as in (d).

(f) Time-lapse reconstructed intensity along the red line in (b) by traditional LFM at *z*=30 µm.

(g) Time-lapse reconstructed intensity along the red line in (c) by DiLFM at *z*=30 µm.

(h) Zoom-in area around the red line in (b) by traditional LFM with time-lapse at *z*=30 µm. The time window is indicated by the dashed box in (f). Arrows mark two blood cells at *z*=30 µm. The lines in (h) are the same as the line in (b).

(i) Zoom-in area around the red line in (c) by DiLFM with time-lapse at *z*=30 µm. The time window is indicated by the dashed box in (g). Arrows mark two blood cells at *z*=30 µm. The lines in (i) are the same as the line in (c).

Scale bars in (b) and (c) are 50 µm, in (d) and (e) are 10 µm, in (h) and (i) are 5 µm.

**Figure S7**

**
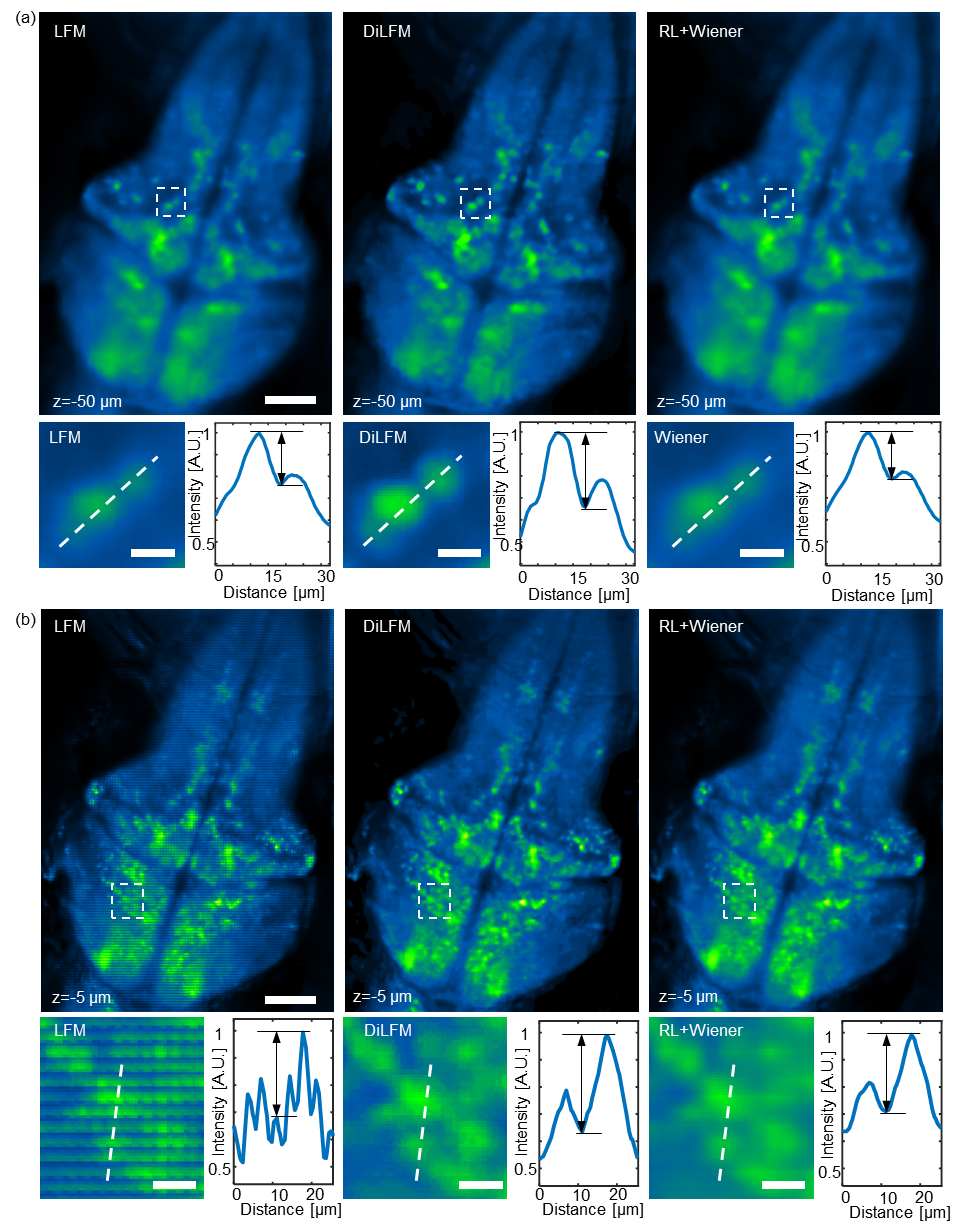
**

**Fig. S7. Reconstruction comparisons between LFM, DiLFM and RL+Wiener filter.**

(a) First row, reconstructions of traditional LFM, DiLFM, and RL with Wiener filters of GCaMP6s labeled zebrafish brain at *z*=-50 µm. The second row, zoom-in panels labeled by white dashed boxes in the first row and intensity profile across the white dashed line. The contrasts of the two neurons are 0.136, 0.112, and 0.205 for traditional LFM, RL+Wiener filter, and DiLFM, respectively.

(b) is the same as (a) but the depth is at *z*=-5 µm. The contrasts of the two neurons are 0.198, 0.17, and 0.227 for traditional LFM, RL+Wiener filter, and DiLFM, respectively.

Scale bars in main panels of (a) and (b) are 100 µm, in zoom-in panels are 10 µm.

**Figure S8**


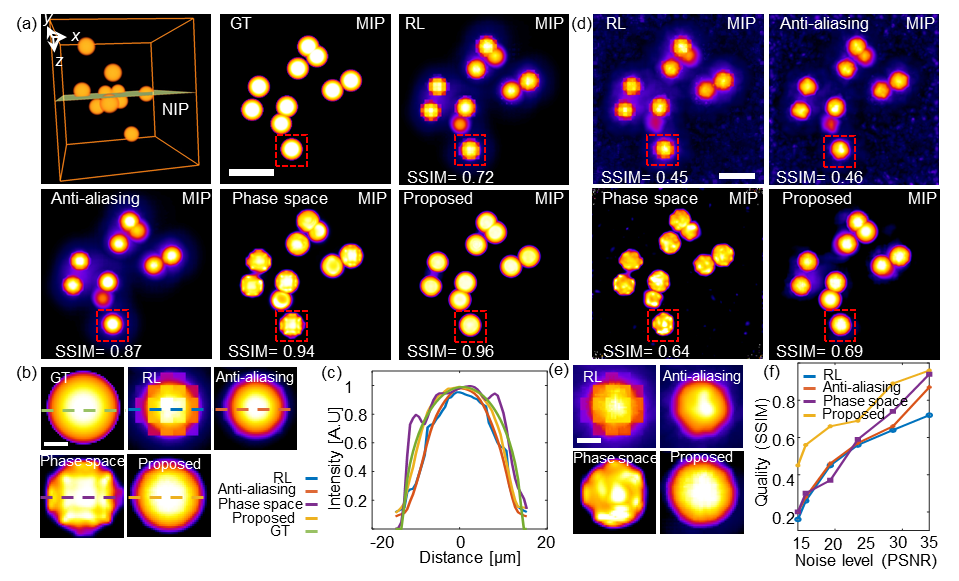


**Fig. S8. Reconstruction quality comparisons among different algorithms over quadradic intensity spheres (**$\boldsymbol{I}\mathbf{=}\sqrt{\mathbf{1-}\boldsymbol{r}^{\mathbf{2}}}$**).**

(a) 3D rendering of the volume with spherical objects and the maximum intensity projections (MIPs) of the ground truth volume (GT) and reconstructed volumes by RL, RL with an anti-aliasing filter, phase space, and DiLFM. Structural similarity index (SSIM) of different approaches are labeled in each image.

(b) Zoom-in panels of one of the spherical objects indicated by the red dashed boxes in (a), which shows DiLFM achieves smooth gradient intensity distribution across the sphere.

(c) Intensity profile along the dashed line in (b).

(d) MIPs of reconstructions by different approaches under mixed Poisson and Gaussian noise (noise level PSNR=24.6).

(e) Zoom-in panels of one of the spherical objects indicated by the red dashed boxes, showing DiLFM achieves the least-distorted result.

(f) SSIM of different reconstructions in noise levels from PSNR=14.5 dB to 34 dB.

Scale bars in (a) and (d) are 50 µm, in (b) and (e) are 10 µm.

**Figure S9**


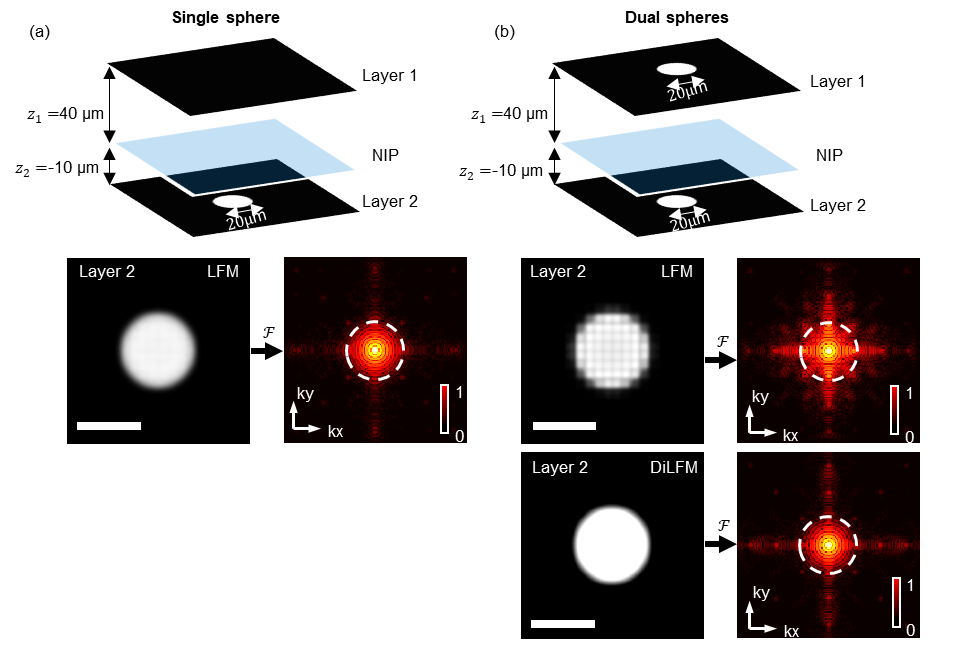


**Fig. S9. DiLFM reduces depth crosstalk artifacts in dual spheres simulations.**

(a) Top, a simulated scene where a 20 µm diameter sphere is put at $z_{1}=-$10 µm underneath the native image plane (NIP). Bottom, traditional LFM reconstruction result at $z_{1}=-$10 µm (left) and its Fourier domain frequency plot in the log scale.

(b) First row, a simulated scene where two 20 µm diameter spheres are put at $z_{2}=+40$ µm and $z_{1}=-$10 µm from NIP. The second row shows the traditional LFM reconstruction $z_{1}=-$10 µm (left) and its Fourier domain frequency plot in the log scale. The third row shows the DiLFM reconstruction at $z_{1}=-$10 µm (left) and its Fourier domain frequency plot in the log scale. The energy of frequency components outside the white dashed ring by DiLFM is 38.2414 times smaller than that by traditional LFM.

Scale bars are 20 µm.

**Figure S10**


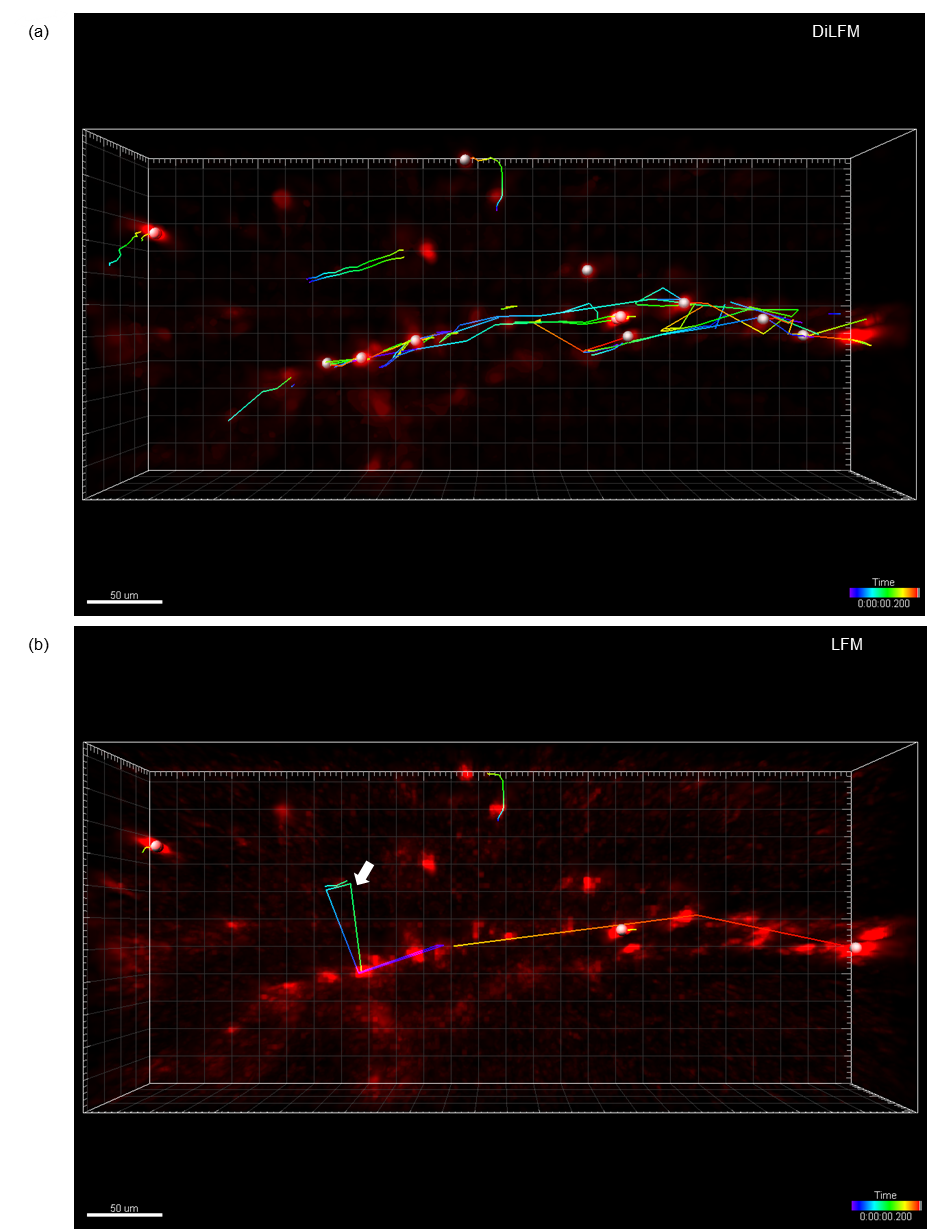


**Fig. S10. Blood-cell tracking through Imaris 9.0.1 software in low-power illumination dosage.**

(a) Cell tracking results of reconstructed zebrafish blood cell by DiLFM, which is the same experiment in Fig.4. The overall tracing time length is 0.2 s. The total number of tracked cells is 37, which is automatically decided by the tracking algorithm in the software. Cell traces are color-coded by different time stamps and tracked cells are overlaid by white spheres.

(b) The same as (a) but by traditional LFM. The total number of tracked cells is 6. Some traces are missing and some are irregular, as marked by the white arrow.

Scale bars are 50 µm. Color bars show different time stamps.

**Figure S11**


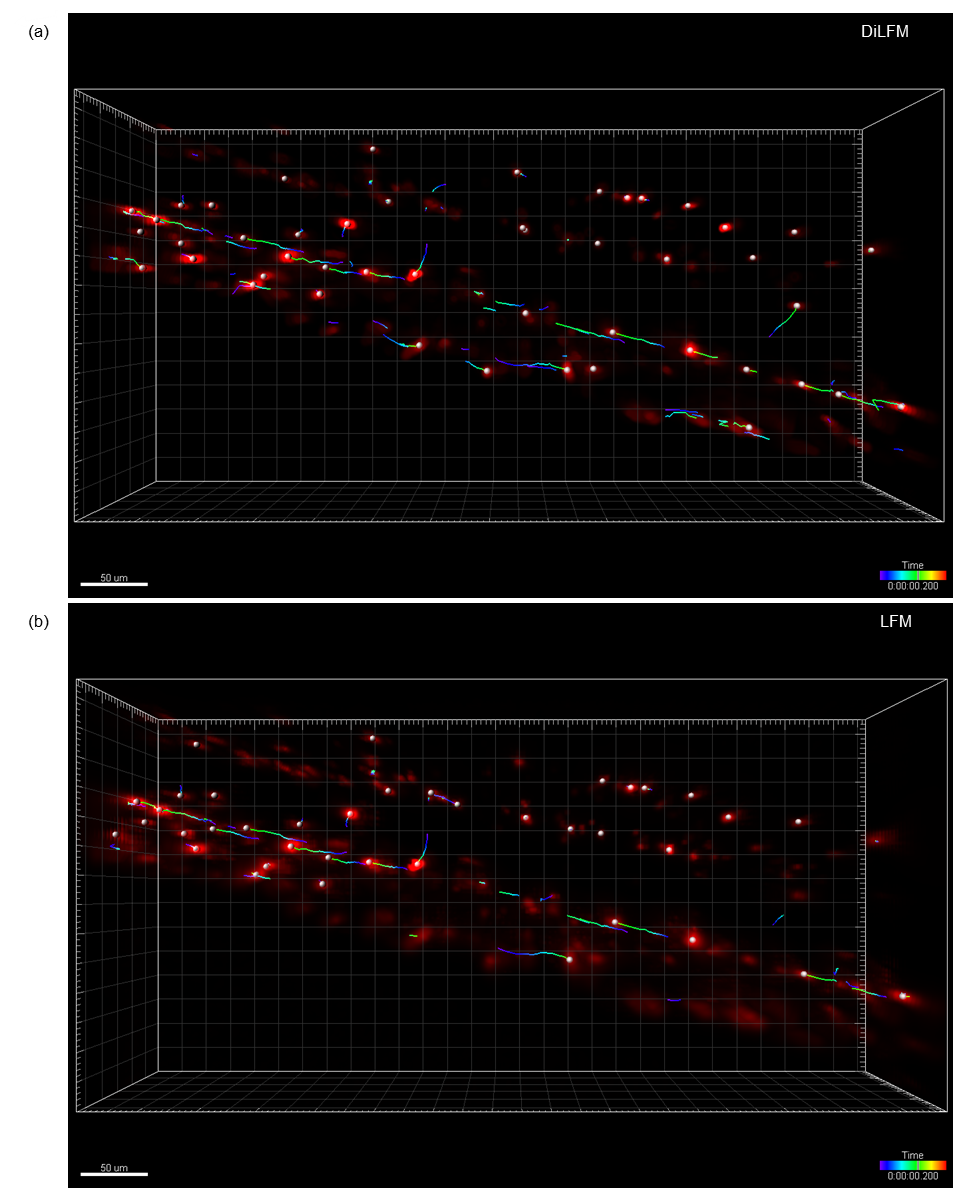


**Fig. S11. Blood-cell tracking through Imaris 9.0.1 software.**

(a) Cell tracking results of reconstructed zebrafish blood cell by DiLFM, which is the same experiment in Supplementary Fig. 6. The overall tracing time length is 0.35 s. The total number of tracked cells is 128, which is automatically decided by the tracking algorithm in the software. Cell traces are color-coded by different time stamps and tracked cells are overlaid by white spheres.

(b) The same as (a) but by traditional LFM. The total number of tracked cells is 99.

Scale bars are 50 µm. Color bars show different time stamps.

**Figure S12**


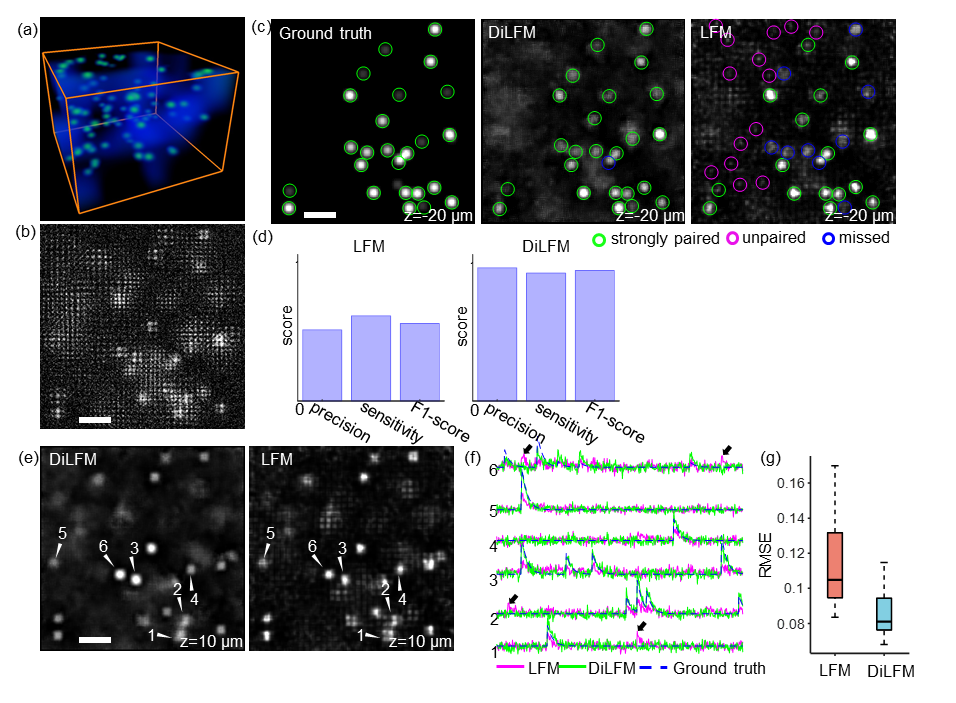


**Fig. S12. DiLFM increases neuron segmentation and calcium activities inferring accuracy.**

(a) 3D rendering of simulated brain tissue with virtual neurons.

(b) LFM capture of neurons in (a) with noise contaminations.

(c) Neuron segmentation results of ground truth data, DiLFM, and traditional LFM. Green circles show strong pairs between ground truth data and the other two methods, magenta circles show unpaired segmentations, and blue circles show missed segmentations compared to ground truths.

(d) Precision, sensitivity, and F1-scores of segmentations in (c) by traditional LFM and DiLFM.

(e) Exemplary reconstructions of one frame at *z*=10 µm by DiLFM (left) and traditional LFM (right).

(f) Neuron activities marked by white arrows in (e). Magenta traces for traditional LFM, green traces for DiLFM, and dashed blue traces for ground truth data. Black arrows mark the wrong activities by traditional LFM

(g) Root Mean Square Error (RMSE) of inferred calcium traces compared to ground truth by traditional LFM (orange) and DiLFM (blue).

Scale bars are 30 µm.

**Table S1**

**Optical objective and illumination source usages in experiments**

| **Figure** | **Objective** | **Illumination source** |
| --- | --- | --- |
| Figure 2 | Zeiss 40x, NA 1.0 objective | 488 nm laser,  27.3$\mathrm{mW}\mathrm{mm}^{-2}$ |
| Figure 3 | Olympus 10x, NA 0.5 objective | Simulation |
| Figure 4 | Zeiss 20x, NA 0.5 objective | 488/561 nm laser,  0.15$\mathrm{mW}\mathrm{mm}^{-2}(488 nm$), 0.12$\mathrm{mW}\mathrm{mm}^{-2}(561 nm)$ |
| Figure 5 | Zeiss 20x, NA 0.5 objective | Mercury lamp,  0.37$\mathrm{mW}\mathrm{mm}^{-2}$ |
| Figures S2-4 | Olympus 10x, NA 0.5 objective | Simulation |
| Figure S5 | Zeiss 40x, NA 1.0 objective | 488 nm laser,  27.3$\mathrm{mW}\mathrm{mm}^{-2}$ |
| Figure S6 | Zeiss 20x, NA 0.5 objective | 561 nm laser, 6.8$\mathrm{mW}\mathrm{mm}^{-2}$ |
| Figure S7 | Zeiss 20x, NA 0.5 objective | Mercury lamp,  0.37 $\mathrm{mW}\mathrm{mm}^{-2}$ |
| Figure S8 | Olympus 10x, NA 0.5 objective | Simulation |
| Figure S9 | Olympus 10x, NA 0.5 objective | Simulation |
| Figure S12 | Zeiss 20x, NA 0.5 objective | Simulation |

**Table S2**

**Dictionary training parameters in experiments**

| **Figure** | **RL iteration number** | **Dictionary size (element number)** | **Patch size (in pixels)** |
| --- | --- | --- | --- |
| Figure 2 | 3 | 1000 | 3 |
| Figure 3 | 2 | 100 | 10 |
| Figure 4 | 3 | 1000 | 10 |
| Figure 5 | 3 | 1000 | 5 |
| Figure S2 | 1 | 1500 | 3 |
| Figure S3 | 3 | 1500 | 3 |
| Figure S4 | 3 | 1500 | 3 |
| Figure S5 | 3 | 1000 | 3 |
| Figure S6 | 3 | 1000 | 5 |
| Figure S7 | 3 | 1000 | 5 |
| Figure S8 | 2 | 100 | 10 |
| Figure S9 | 2 | 100 | 10 |
| Figure S12 | 3 | 1000 | 5 |

**Note S1:** Sparse representation of high- and low-fidelity pairs

In DiLFM, we assume that the low- and high-fidelity patches in our DiLFM share the same sparse representation coefficients since we assume the artifact contamination and the resolution blur in LFM reconstructions are approximately linear operators. In the following we first prove that the linearity of the artifact contamination operator and resolution blur operator can derive the same sparse representation coefficients of low- and high-fidelity patches, then we provide evidence to support the linearity of the artifact contamination and blur operators in LFM.

If we denote the artifact contamination operator as $A$, and the blur operator as $B$, then the relationship between low-fidelity patch $\hat{I}_{j}^{ref}$and high-fidelity patch $I_{j}^{ref}$ can be denoted as

$$\begin{aligned} \hat{I}_{j}^{ref}=ABI_{j}^{ref}\#\left( S1 \right) \end{aligned}$$

We firstly denote the high-fidelity patch $I_{j}^{ref}$ can be represented as

$$\begin{aligned} I_{j}^{ref}=I_{j}^{h,ref}+\hat{I}_{j}^{ref}=D_{h}\beta+\hat{I}_{j}^{ref}\#\left( S2 \right) \end{aligned}$$

i.e. a high-fidelity patch can be sparsely represented through the high-fidelity dictionary $D_{h}$ with coefficient $\beta$. Then we can get

$$\begin{aligned} \hat{I}_{j}^{ref}=ABI_{j}^{ref}=AB\left( D_{h}\beta+\hat{I}_{j}^{ref} \right)=ABD_{h}\beta+AB\hat{I}_{j}^{ref}\#\left( S3 \right) \end{aligned}$$

which directly yields that $\hat{I}_{j}^{ref}=\left( I-AB \right)^{-1}ABD_{h}\beta=D_{l}\beta$, i.e. low-fidelity patch $\hat{I}_{j}^{ref}$ can be sparsely represented by $D_{l}$ with the same coefficient $\beta$. The above proof shows that the low- and high-fidelity components of the $I_{j}^{ref}$ share the same sparse representation under the assumption of linear blur operator $B$ and artifacts contamination operator $A$. Next, we discuss the validation of the linear assumption about artifacts and blur operators.

1) Artifacts contaminator operator $A$ is approximately linear

We discuss three different artifacts by traditional Richardson Lucy (RL) iteration in the manuscript. The ringing artifacts are raised by too many RL iterations. In our DiLFM, we use few iterations of RL, which means the $A$ does not contain such an artifact model. The blocking artifacts happen in the native image plane (NIP) which can be represented as the nearest down-sampling step, thus it is a linear operator. The depth crosstalk artifacts result in grid-like patterns as shown in Supplementary Fig. 4, which can be approximately represented by frequency contamination

$$\begin{aligned} \hat{I}_{j}^{ref}=\mathcal{F}^{-1}\left\{ \mathcal{F}\left\{ I_{j}^{ref} \right\}\cdot Q \right\}\#\left( S4 \right) \end{aligned}$$

where $Q$ is frequency-domain contamination matrix which amplifies specific frequency components and $\mathcal{F}$ is Fourier transformer. Since $\mathcal{F}$ can be represented in matrix form and $Q$ is relatively fixed for the same kind of samples, the relationship in Eq. (S4) is also approximately linear, which means depth crosstalk contamination is an approximately linear operator. Thus, artifact contamination operator $A$ is approximately linear.

2) Blur operator $B$ is linear

The blur operation can be represented as a low-pass filter which is a linear model, thus blur operator $B$ is linear.

Through the above discussion, we validate that both the artifact contamination operator $A$ and the blur operator $B$ are approximately linear across similar kinds of samples, thus low- and high-fidelity components of the $I_{j}^{ref}$ share the same sparse representation as we derive in Eq. (S3).
